# From the Cochrane Library: Interventions for Chronic Pruritus of Unknown Origin

**DOI:** 10.2196/53271

**Published:** 2024-08-21

**Authors:** Pritika Parmar, Amit Singal, Mindy D Szeto, Gaurav N Pathak, Viktoria Taranto, Thu M Truong, Babar Rao, Andrea Andrade Miranda, Juan VA Franco, Robert P Dellavalle

**Affiliations:** 1 Department of Dermatology University of Minnesota Medical School Minneapolis, MN United States; 2 Rutgers New Jersey Medical School Newark, NJ United States; 3 Department of Dermatology Rutgers Robert Wood Johnson Medical School New Brunswick, NJ United States; 4 New York Institute of Technology College of Osteopathic Medicine Glen Head, NY United States; 5 Department of Dermatology Weill Cornell Medicine New York, NY United States; 6 Department of Dermatology Hospital Italiano de Buenos Aires Buenos Aires Argentina; 7 Institute of General Practice Heinrich Heine University Düsseldorf Germany

**Keywords:** Cochrane, systematic review, randomized controlled trial, RCT, pruritus, chronic pruritus, chronic pruritus of unknown origin, CPUO, serlopitant, dupilumab, pregabalin

## Introduction

Chronic pruritus of unknown origin (CPUO) is characterized by pruritus lasting longer than 6 weeks and is a diagnosis of exclusion with no identifiable cause; the estimated prevalence ranges between 7% and 45.9% and is the highest in the older population [1]. Affected individuals experience a significant disruption to quality of life, including sleep disturbances and psychological concerns, which can further contribute to itching [2]. Treatment of patients with CPUO is particularly challenging due to its unclear pathophysiology [3]. No Food and Drug Administration–approved treatment for CPUO currently exists. First-line treatment can consist of antihistamines and topical steroids; unfortunately, treatments for CPUO show only variable responsiveness [4]. Research on interventions for CPUO is sparse, including assessments of safety and efficacy. A Cochrane systematic review, “Interventions for chronic pruritus of unknown origin,” assessed interventions for CPUO in adults and children by examining the available evidence from randomized controlled trials (RCTs) and quasi-RCTs for the efficacy of CPUO interventions [2].

## Methods

A total of 7148 records published up to July 2019 were obtained in a literature search with only 1 eligible RCT meeting the Cochrane review’s inclusion criteria based on participant population, study design, and interventions [2]. The included multicenter RCT’s participants (N=257) had a 6-week minimum complaint of pruritus unresponsive to first-line treatment and ≥7 cm on the visual analog scale (VAS) at baseline, which is considered to indicate a severe case of chronic pruritus. The RCT’s exclusion criteria were based on serum creatinine, aspartate aminotransferase, or alanine aminotransferase levels >2 times the upper limit of the reference range or previous diagnoses suggestive of secondary pruritus causes. The RCT quantified the therapeutic impact (via percentage change in VAS) of 3 different dosing levels of serlopitant, a novel neurokinin 1 (NK1) receptor antagonist that acts to inhibit the NK1-mediated itch signaling pathway. The primary endpoints of the included RCT were evaluation of VAS itch severity and adverse events. Secondary endpoints considered by the Cochrane search included health-related quality of life, sleep disturbances, depression, and patient satisfaction. The GRADE (Grading of Recommendations, Assessment, Development, and Evaluations) approach was applied to interpret the certainty of the RCT findings [5].

## Results

The primary and secondary outcomes of the RCT are summarized in Table 1.

Compared to placebo, patients who received 5 mg serlopitant orally once daily for 6 weeks showed significant improvements in VAS (relative risk [RR] 2.06, 95% CI 1.27-3.35) and reduced patient-reported numerical rating scale (NRS) itch intensity (mean difference –10.30, 95% CI –20.01 to –0.59); the number needed to treat was approximately 4. The potential for an increased risk of adverse events was unclear (RR 1.48, 95% CI 0.87-2.50). According to the GRADE assessment, the certainty of the evidence was low to very low, with risk-of-bias concerns due to missing outcome data and presence of potential underlying diagnoses in many RCT participants. Depression and patient satisfaction were not addressed in this RCT.

**Table 1:** Primary and secondary endpoint findings compared to placebo in a randomized controlled trial evaluating different doses of serlopitant for chronic pruritus.

Serlopitant dose^a^	Primary endpoints		Secondary endpoints	
	Reduction (≥4 cm) in VAS^b^, RR^c^ (95% CI)	Adverse events^d^, RR (95% CI)	Quality of life^e^, MD^f^ (95% CI)	Sleep disturbance^g^, RR (95% CI)
0.25 mg (N=64)	1.66 (1.00 to 2.77), n=127^h^	1.29 (0.75 to 2.24), n=127	–5.70 (–13.18 to 1.78), n=127	0.60 (0.31 to 1.17), n=127
1 mg (N=65)	1.50 (0.89 to 2.54), n=126	1.45 (0.86 to 2.47), n=128	–6.90 (–14.38 to 0.58), n=128	0.38 (0.17 to 0.84), n=128
5 mg (N=64)	2.06 (1.27 to 3.35), n=126	1.48 (0.87 to 2.50), n=127	–4.20 (–11.68 to 3.28), n=127	0.49 (0.24 to 1.01), n=128

^a^All doses (including the placebo) were administered orally once a day for 6 weeks.

^b^VAS: visual analog scale (range 0-10 cm).

^c^RR: relative risk.

^d^Adverse events were defined as the number of participants with any adverse event.

^e^Health-related quality of life was assessed with the Dermatology Life Quality Index score (range 0-30). A higher score indicates greater impairment" so it's clear these are quality of life improvements.

^f^MD: mean difference.

^g^Sleep disturbances were defined as the number of participants with insomnia (assessed with the Pittsburgh Sleep Symptom Questionnaire).

^h^n=total number of participants included in the analysis (placebo+serlopitant groups).

## Discussion

Findings from smaller-scale studies conducted after publication of the Cochrane review suggest that new therapeutic approaches, including pregabalin and dupilumab, may be more effective at reducing VAS and NRS scores in patients with treatment-resistant CPUO [3,4]. Pregabalin is considered to alleviate CPUO through modulating thresholds of the C-fibers shown to transmit itch signals by suppressing the release of several neurotransmitters such as substance P, which may be chronically elevated in patients with CPUO [4]. Dupilumab, which can inhibit interleukin (IL)-4 and IL-13, with well-known anti-inflammatory properties, may help to alleviate CPUO through cytokine-neural interactions [3]. Pregabalin decreased VAS scores for 70% of patients with CPUO refractory to antihistamine therapy [4]. Likewise, treatment with dupilumab resulted in a substantial mean decrease in the NRS itch score by 7 [3]. These findings suggest potential alternative treatment approaches for patients who have treatment-refractory CPUO, which remains a diagnosis of exclusion with unclear etiology. Current treatments, including emollient creams, cooling lotions, topical corticosteroids, topical antidepressants, systemic antihistamines, systemic antidepressants, systemic anticonvulsants, and phototherapy, lack extensive study, especially in RCTs [2].

**Figure 1:**
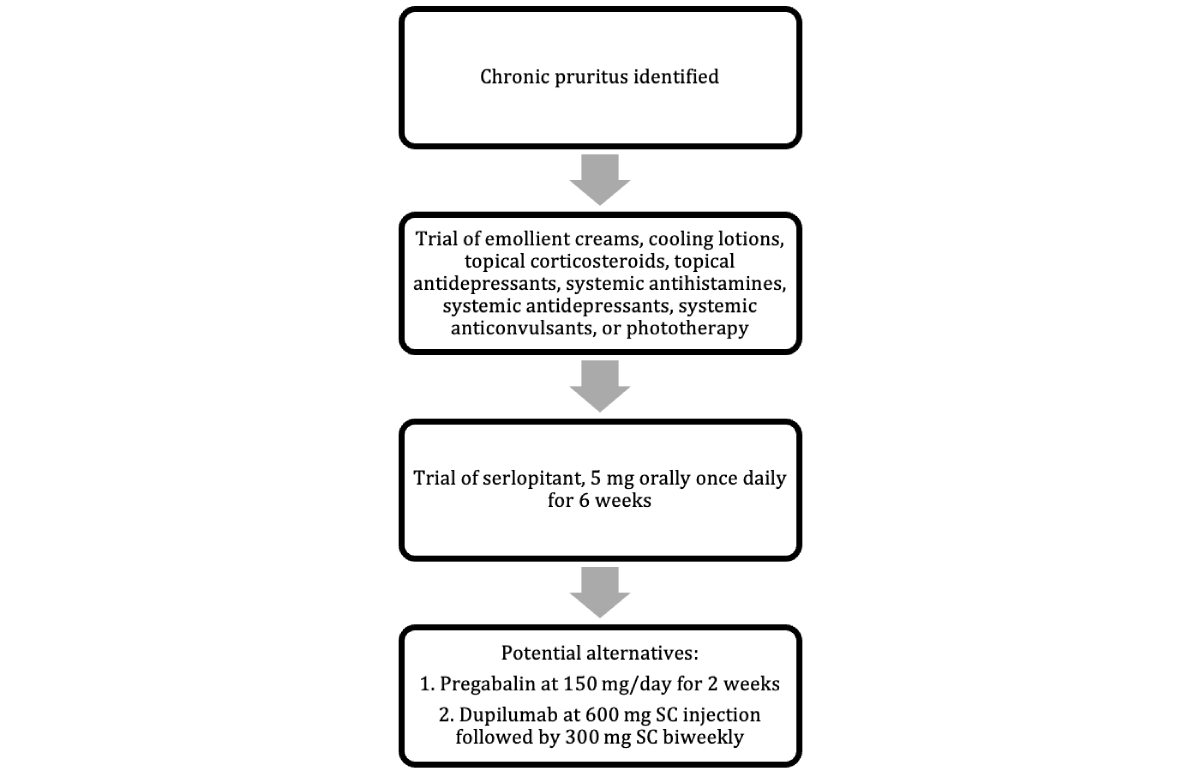
Practical algorithmic treatment options once chronic pruritus is identified in a patient based on current treatment approaches and recent studies. SC: subcutaneous.

Taken together, these studies suggest that after current treatment approaches fail, serlopitant (5 mg orally once daily for 6 weeks), pregabalin (150 mg daily for 2 weeks), or dupilumab (600 mg subcutaneous injection followed by 300 mg subcutaneous injection biweekly) are potential treatment options for CPUO (Figure 1).

Poorly understood pruritic cutaneous manifestations related to COVID-19, along with the frequent handwashing, personal protective equipment use, and psychosocial stress during the pandemic, have presented difficulties in determining the root causes of itch in many patients, likely exacerbated by reduced access to health care and a heightened fear of infection [6]. Postpandemic recovery may require further research to reconsider ideal CPUO management approaches given interruptions to care; ultimately, additional investigation is needed to characterize the various molecular underpinnings of CPUO and may aid in more effective and targeted therapeutics.

## Abbreviations

CPUO: chronic pruritus of unknown origin
GRADE: Grading of Recommendations, Assessment, Development, and Evaluations
IL: interleukin
NK1: neurokinin 1
NRS: numerical rating scale
RCT: randomized controlled trial
RR: relative risk
VAS: visual analog scale
